# Retraining gait in Parkinson’s Disease via a personalised app: A study protocol

**DOI:** 10.1371/journal.pone.0346508

**Published:** 2026-04-15

**Authors:** Conor Wall, Peter McMeekin, Victoria Hetherington, Rosie Morris, Rodrigo Vitorio, Richard Walker, Alan Godfrey

**Affiliations:** 1 School of Computer Science, Northumbria University, Newcastle upon Tyne, United Kingdom; 2 School of Healthcare and Nursing Sciences, Northumbria University, Newcastle Upon Tyne, United Kingdom; 3 Cumbria Northumberland Tyne and Wear NHS Foundation Trust, Newcastle upon Tyne, United Kingdom; 4 School of Sport, Exercise and Rehabilitation, Northumbria University, Newcastle upon Tyne, United Kingdom; 5 Northumbria Healthcare NHS Foundation Trust, North Shields, United Kingdom; Pennsylvania State University Main Campus: The Pennsylvania State University - University Park Campus, UNITED STATES OF AMERICA

## Abstract

Parkinson’s disease (PD) is a progressive neurological disorder that often leads to gait impairments and an increased risk of falls, negatively impacting quality of life. Inertial-based wearables have extended gait evaluation beyond clinical settings, but their technical integration and general accessibility hinder widespread adoption. Smartphones, equipped with inertial sensors, present a scalable approach for gait assessment and retraining/rehabilitation. Auditory cueing, specifically music, has shown promise to retrain gait, yet its use and uptake is often limited by a lack of personalisation. This paper outlines an exploratory and experimental protocol to evaluate CuePD, a smartphone application/app that delivers near real-time gait assessment and personalised auditory cues (including music) for gait retraining in people with PD (PwPD). The protocol characterises beneficial and non-beneficial responses to different cueing strategies and presents separate analysis for the technical validation and gait response outcomes to reduce interpretive ambiguity between measurement performance and behavioural effects. Specifically, the protocol presents: 1) validation of CuePD’s spatio-temporal gait characteristics in a controlled laboratory setting; 2) process to understand the usefulness of various auditory cues (metronome beats, instrumental music, and vocal music) to improve gait stability (i.e., increase gait speed and stride length while reducing gait variability); 3) evaluation of acceptance and preference of PwPD for personalised music cues; and 4) relationship between PwPD's musicality and their ability to adhere to auditory cues. Findings from the study will inform the suitability of gait retraining via smartphone-based app technology, personalised to the individual. Personalisation (gait characteristics and music) may potentially encourage long-term engagement for gait retraining which could lead to reduce falls and improved quality of life for PwPD. By explicitly accounting for individual variability, this protocol aims to clarify for whom and under what conditions auditory cueing is appropriate. Future work should systematically evaluate this approach as responses to auditory and/or music-based cueing in PwPD are heterogeneous with possible neutral or adverse effects, e.g., cognitive overload or exacerbation of freezing. This trial is registered at ClinicalTrials.gov (NCT06941779).

## Introduction

People with a neurological disorder have a heightened fall risk [1] which can increase morbidity and mortality rates [2]. Increased fall risk often equates to a decline in an individual's confidence to perform routine everyday tasks, which increases the likelihood of future falls, perpetuating a cycle of apprehension and susceptibility [3,4]. That leads to many individuals struggling to actively participate in communities, diminishing their quality of life [5,6]. Parkinson's disease (PD) is a progressive neurological disorder and has the fastest-growing incidence rate, with prevalence projected to double over the next 30 years, with 10 million people with PD (PwPD) living worldwide [7,8]. PD adversely impacts mobility and increases fall risk through gait disturbances, with increased stride-to-stride variability being directly related to an increased fall rate [9]. Over 60% of PwPD encounter at least one fall annually, of which 39% report recurrent falls, varying from 4.7 to 67.6 falls/person/year, with an average of 20.8 falls overall [10]. To reduce fall risk, rehabilitation is administered through walking/gait retraining stemming from a gait assessment in a laboratory/lab or clinic [11,12].

Traditional gait assessment to inform retraining stems from a visual analysis of an individual's overall gait pattern, where tacit expertise can help to identify subtle gait disturbances [13]. However, visual observation alone is subjective and creates inconsistencies between assessors [14] as well as within and across assessments [15]. Although lab-based technologies (e.g., instrumented walkways) enable higher-resolution data capture to provide more objective insights [16,17], they are expensive and limited to bespoke settings which prohibits widespread use [18]. Accordingly, delivery of care and related services to those with increased fall risk is restricted [19]. There is a need to decentralise care by adopting pervasive and scalable technologies for assessment and retraining where wearable inertial measurement units (IMUs) have been suggested [20,21] as they have demonstrated effectiveness in quantifying gait characteristics [22–27]. Regardless, wearable IMU systems may still be described as expensive and technically cumbersome, limiting widespread availability [28]. To enable cost-effective and widespread use, contemporary smartphones (routinely equipped with inertial sensors) are a scalable and practical alternative [29,30].

Although smartphones may be equipped with inertial sensors, they also host a plethora of other technologies which could be harnessed within a gait retraining paradigm. For example, smartphones are readily equipped with wireless communication capabilities, cameras, high-resolution displays, and speakers. The latter is key to deliver retraining via auditory cueing, an effective method for achieving significant gait improvements [31,32], compared to alternatives such as treadmill therapy [33–36]. Auditory cueing typically utilises the rhythmic beat of a metronome to assist individuals in maintaining a steady walking pace [37]. Nevertheless, users often find metronomes to be monotonous and tedious, leading to a lack of long-term engagement [38–40]. Alternatively, music cueing offers a more engaging experience, while also exhibiting a positive influence on gait within PwPD [41–43]. However, the routine uptake of music cueing can be limited by a lack of personalisation, where an individual’s condition and music preferences are not considered, potentially leading to suboptimal engagement and efficacy [44]. Therefore, the development of personalised cueing in PwPD to better retrain gait via a scalable smartphone-based gait assessment is necessary to help reduce fall risk [45–47].

This protocol outlines the methodology for examining CuePD, a smartphone application/app that provides near real-time gait assessment to deliver personalised auditory cues for gait retraining in PwPD. It primarily focuses on determining gait improvements from increases in stride length, gait speed, cadence and reductions in gait variability. Secondly, it will determine the validity of the app’s robustness in PwPD, measuring clinically relevant gait characteristics including mean, variability, and asymmetry across temporal, spatial, and spatiotemporal characteristics. Equally, this protocol also aims to assess usability and relationship between a PwPD musicality, i.e., their knowledge, training, and interest in music, and their ability to adhere to auditory cues.

Therefore, the primary objectives of this protocol are to (i) validate gait characteristics from CuePD and (ii) evaluate the effects of personalised auditory cues (metronome beats, instrumental music, and vocal music) to retrain and improve gait in a lab, i.e., increase gait speed, cadence and stride length while reducing gait variability. Our secondary objectives are to assess recruited PwPD and their (a) acceptance and preference of personalised music cues compared to traditional metronome cueing while (b) understanding their ability to adhere to cues due to their musicality.

## Materials and methods

### Study design

This is a crossover interventional design study conducted in a controlled gait laboratory setting. It aims to assess and validate participants’ responses to different auditory cues, such as metronome beats, instrumental music, and vocal music, while walking under controlled conditions. The lab environment allows for precise measurements of gait characteristics, providing detailed insights into the use of personalised auditory cues in regulating the gait of PwPD. As this is a study protocol, no data have been included, and it conforms to PLOS data policy.

### Ethical approval

An ethics application (Ref: 3231) was submitted to Northumbria University research internal ethics committee and approved (06-03-2023). The study received NHS research ethics committee approval (REC, Ref: 24/PR/0684) via the Integrated Research Application System (IRAS, Ref: 327241) and a Health Research Authority (HRA) letter of approval, 25 July 2024. The authors confirm that all ongoing and related trials for this intervention are/will be registered. This trial was listed on the ClinicalTrials.gov registry with study ID NCT06941779 on 24 April 2025. The trial team was unaware of the requirement to register prior to participant recruitment and completed registration as soon as this oversight was recognised.

### Participants

Sixty (60) PwPD will be recruited for this study, with no specific criteria for recruitment regarding gender or sex.

### Recruitment

Participants will be recruited through the Dementias & Neurodegenerative Diseases Research Network (DeNDRoN) Research Case Register, which includes PwPD with confirmed diagnoses interested in local research. Additionally, participants may be identified through the movement disorders clinics within Northumbria Healthcare NHS Foundation Trust. Interested participants will be provided with a REC approved Participant Information Sheet. Recruitment opened in September 2024 and will terminate in December 2025 where results are expected in February 2026. Before participating in the study, written, informed consent will be obtained from all participants. Table 1 outlines the inclusion and exclusion criteria for the study. No hearing inclusion criterion (e.g., whisper test) is included because the intervention (CuePD) delivers adjustable-volume rhythmic cues via headphones/speakers, enabling participants with mild hearing impairment to perceive and respond to the beat. Excluding such individuals would unnecessarily limit recruitment and reduce generalisability, while the ability to perceive speech at 2 m is not directly relevant to rhythm entrainment for gait training.

**Table 1:** Study inclusion and exclusion criteria.

Inclusion criteria	Exclusion criteria
• Aged ≥50 years • Able to walk unaided. • Diagnosis of idiopathic PD, as defined by the UK Brain Bank criteria. • Score ≥21/30 on Montreal Cognitive Assessment (MoCA) which is used to classify non-demented PD (PD dementia is < 21/30).	• Non-English speakers • History of stroke, traumatic brain injury or other neurological disorders (other than PD) • Acute lower back or lower extremity pain, peripheral neuropathy, and musculoskeletal disorders that would affect tasks. • Unstable medical condition including cardio-vascular instability in the past 6 months • Unable to comply with the testing protocol or currently participating in another interfering research project

### Equipment

**Fig 1:**
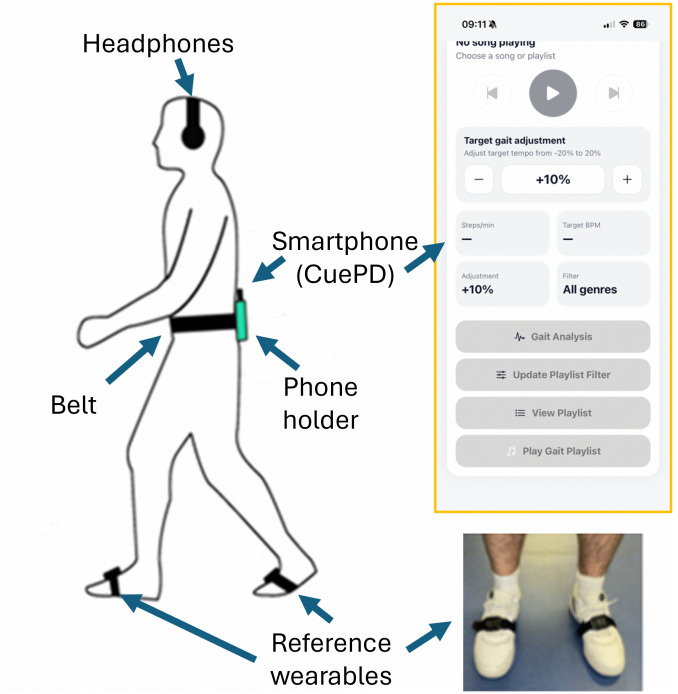
Equipment Configuration for the Study. This figure displays the setup of the smartphone device with CuePD installed, reference IMU wearables, a pair of wireless headphones, a belt, and a phone holder as used in the experiments.

The equipment used in this study includes a (i) smartphone with the CuePD app installed, (ii) reference IMU wearable, (iii) a pair of wireless headphones and (iv) a belt attachment with phone holder. The placement and orientation of these devices are illustrated in Fig 1.

#### Reference standard.

The reference for this study is Opal (https://clario.com/solutions/precision-motion-for-research/) which interfaces with Mobility Lab, a validated commercially available system that enables comprehensive gait assessment [22]. It allows for the wireless synchronisation of up to six IMUs (Opal V1), which incorporate tri-axial accelerometers (range:  ±  16g and  ±  200g), gyroscopes (range:  ±  2000°/s), and magnetometers (range:  ±  8 Gauss). During each walking task, a single Opal IMU (26g, 50 × 40 × 14 mm), sampling data at 128 Hertz/Hz, will be attached to the talus joint of each foot of the participant. Reference standard data will be used to validate CuePD’s temporal, spatial, and spatiotemporal gait characteristics (inc. asymmetry and variation) in PwPD to ensure primary outcomes are robust.

#### Smartphone and CuePD.

CuePD will be installed on an Apple iPhone XS (177g, 143.6 × 70.9 × 7.7 mm), which has embedded tri-axial accelerometer and gyroscope inertial sensors, sampling data at ±8 g and 100 Hz. The smartphone will be securely attached to the participants’ lower back at the 5th lumbar vertebra (L5) using a belt strap and phone holder. The app, built in React Native with JavaScript, uses the API ‘react-native-sensors’ to capture tri-axial accelerometer data at a frequency of 100 Hz, providing high-resolution motion data. It includes a countdown for data recording, initiated by a user prompt, and after each one-minute session, it transmits the data to a Python server on Azure through a RESTful API using HTTP POST requests. This server, deployed on an Azure virtual machine running Ubuntu 20.04, utilises Flask to handle incoming requests and process the gait characteristics using NumPy [48], SciPy [49], and PyWavelets [50] libraries for signal processing. The processed data is stored in a PostgreSQL database hosted on Azure, ensuring reliable data management and retrieval. The server then sends the calculated gait characteristics back to the phone through the same RESTful API.

All audio files for each cueing mechanism are stored as. MP3 files and hosted on Azure Blob Storage, ensuring scalability and durability. When the user initiates cueing on the app, the server retrieves the audio files and estimates the beat per minute (BPM) of the audio file using Librosa, an audio signal analysis library in Python [51]. The server then adjusts the tempo of the audio using the FFmpeg Python library, which provides a reliable time-stretching algorithm [52]. This adjustment aligns the music's beats-per-minute with the user's steps-per-minute (SPM) plus an additional 10%, which has been found effective in promoting longer strides, increasing gait speed, and reducing variability [53–59]. The processed audio file is then re-encoded as. MP3, saved back to the Azure Blob Storage and then sent to CuePD for playback. All participants will wear headphones, cleaned between participants, and comfortably placed on their head over their ears to listen to cueing modalities. For more details on CuePD, read here [60–62].

### Data collection

**Fig 2:**
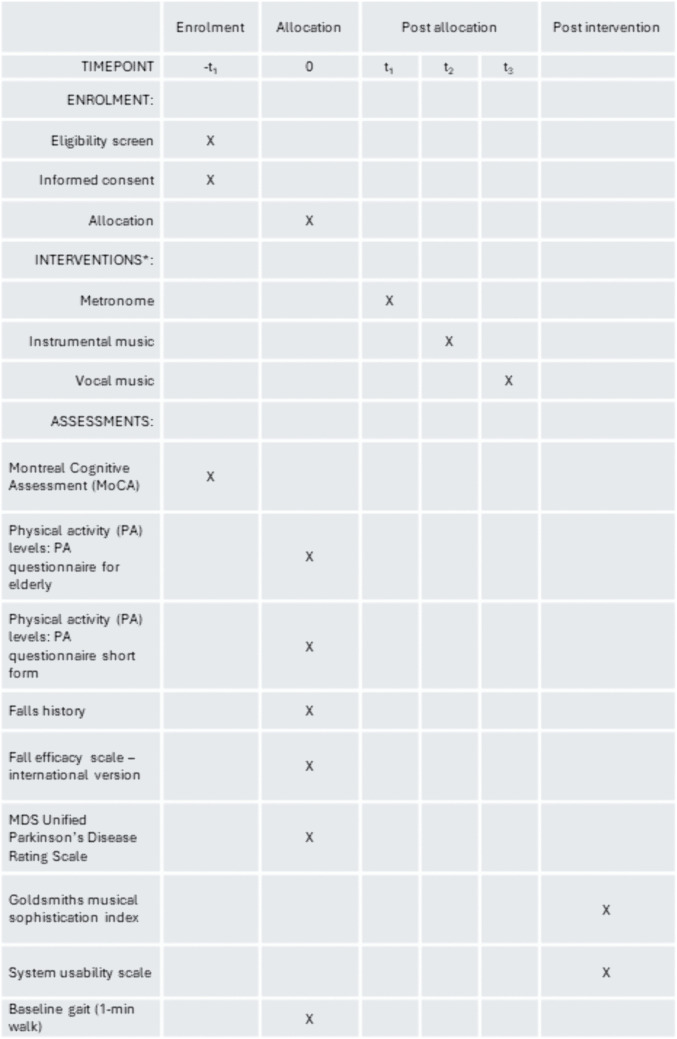
SPIRIT schedule of enrolment, interventions, and assessments. *Order of cued walks are allocated at enrolment.

All testing will be conducted at the Clinical Gait Laboratory, Coach Lane Campus, Northumbria University, Newcastle upon Tyne. Fig 2 outlines the SPIRIT schedule of enrolment, interventions and assessments. Upon attending the gait lab informed written consent procedures will be undertaken, and then participants will be required to answer questionnaires as follows (with i to vii pre and viii to ix post-gait tasks):

(i) **Montreal Cognitive Assessment (MoCA)**: Administered as a screening tool to exclude those with cognitive impairment with <21 being a cut-off point for PwPD. The MoCA will be used as a standardised neuropsychological test acknowledged as a valid and efficient tool for the rapid screening of global cognitive dysfunction [63]. It is used to examine diverse cognitive domains including attention and concentration, executive functions, memory, language, visuo-constructional skills, conceptual thinking, calculations, and orientation.
(ii) **Participant Demographics:** (a) Height and weight, (b) age, (c) ethnicity, (d) duration of PD diagnosis.
(iii) **Education level**: (a) pre/post-high school, (b) college or (c) university
(iv) **Physical activity levels**: To concisely capture physical activity across a range of habitual tasks, two questionnaires will be used: (i) a physical activity questionnaire for the elderly with a reference period of 1-year was used to understand functional household (e.g., cleaning) and community-based activities (e.g., shopping [64]), and (ii) the International Physical Activity Questionnaire Short Form (IPAQ-SF), to assess the types of intensity of physical activity and sitting time to estimate total physical activity in MET-min/week and time spent sitting [65].
(v) **Falls history**: Falls incidence, cause, location and consequences in the previous 12-months will be assessed by a questionnaire based on recommendations [66,67], that leads with the question “In the past year, have you had any fall including a slip or trip in which you lost your balance and landed on the floor or ground or lower level?” [68].
(vi) **Falls Efficacy Scale – International Version (FES-I)**: Used to measure fear of falling in the PwPD [68]. This short, validated measure will assess fear of falling during both basic and demanding activities (physical and social) using a scale from 1 (Not at all concerned) to 4 (Very concerned) across 16 scenarios.
(vii) **MDS-Unified Parkinson's Disease Rating Scale (MDS-UPDRS)**: Commonly employed to evaluate both motor and non-motor symptoms associated with PD [69]. This scale consists of four main sections, each targeting a specific area of evaluation. The first part examines non-motor experiences that occur during daily living, while the second part assesses motor experiences during daily living. The third part evaluates motor examination, while the fourth part focuses on assessing complications related to therapy. To determine the severity of the symptoms, the UPDRS is scored based on a total of 195 points, where higher scores suggest increasing levels of disability.

After completion of all walks, participants will complete:

(viii) **Goldsmiths Musical Sophistication Index (Gold-MSI)**: A 39-item (scored from 1 to 7) self-report designed to assess musicality across five subscales: active engagement (AE), perceptual abilities (PA), musical training (MT), singing abilities (SA), and emotional response (ER) to music [70]. This tool evaluates and provides the detailed extent and nature of musical experiences and skills, but also provides a broader score on musicality, General Music Sophistication (GMS), ranging from 32 to 126. For this study, the scale will be used to assess if there was a relationship between an individual’s general music sophistication and their adherence to the beat of the music cues. Furthermore, the relationship between an individual’s subscale results and beat adherence will also be assessed.
(ix) **System Usability Scale (SUS):** This will evaluate perceptions of the app’s usability, complexity, and overall satisfaction [71]. The SUS produces a composite score that ranges from 0 to 100, calculated by first adjusting the responses from a set of ten questions. For odd-numbered questions, the process involves subtracting one from each score, whereas for even-numbered questions, the score is subtracted from five, resulting in adjusted values that fall between 0 and 4. These adjusted values are subsequently summed together, and the total is multiplied by 2.5 to derive the final SUS score, with higher scores reflecting superior usability.
(x) **Cueing preference:** Lastly, participants are asked a question to determine preferred cueing modality, “Which cueing do you prefer, was it metronome, instrumental music or vocal music?”

#### Walking/gait tasks.

**Fig 3:**
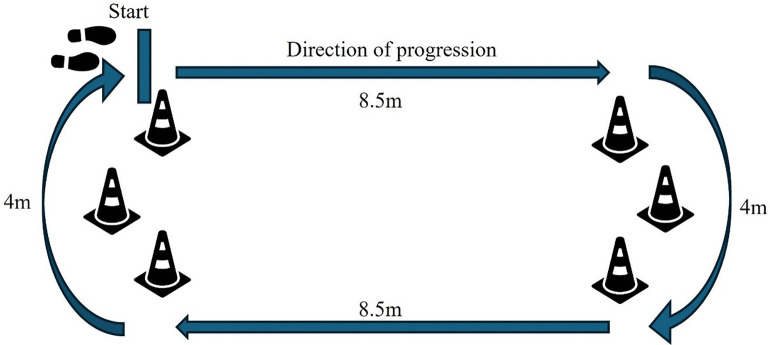
25m walking loop consisting of two straight and two curve sections.

Participants will choose from a selection of music tracks that span genres including pop, rock, R&B, and country. All walks will be 1-minute in duration in a continuous 25m loop, including two 8.5m straight segments and two 4m turns, Fig 3. Walk #1 will be at the participant’s natural walking pace to calculate a baseline cadence, i.e., a walk with no cueing (NC). Walks #2, #4, and #6 will be cued, with external auditory stimuli (BPM set at +10% of baseline cadence) which are metronome (Me), instrumental music (IM), and vocal music (VM). Participants will be assigned to one of three counterbalanced cue sequences for Walks #2, #4, and #6. For example, the first participant enrolled to the study follows Sequence 1, the second follows Sequence 2, the third follows Sequence 3, and the fourth cycles back to Sequence 1, etc.

Following Walks #2 and #4, a two-part washout (WO1 and WO2) will be implemented to minimise any potential carryover effects from the previous auditory cue. Part 1 (of WO1 and WO2) will involve participants sitting and counting backwards in their heads from 30 to 0 in increments of 1 to disengage any psychological responses triggered by the cueing modality [72]. Part 2 (of WO1 and WO2) will consist of a 1-minute walk at the participant’s natural pace, to further reset their gait but also to assess whether any carryover effect remains before the next cue begins. This approach ensures each cue’s effects are evaluated independently of preceding sequences:

- Sequence 1–20 participants: NC, Me, WO1, IM, WO2, and VM.
- Sequence 2–20 participants: NC, IM, WO1, VM, WO2, and Me.
- Sequence 3–20 participants: NC, VM, WO1, Me, WO2, and IM.

**Fig 4:**
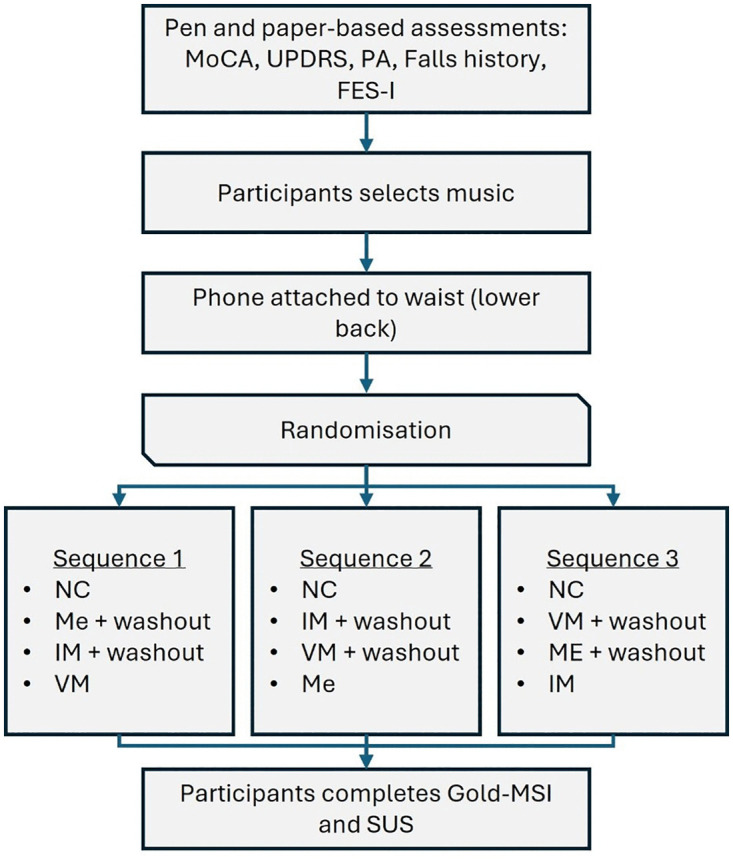
A diagrammatic representation of the study sequence.

Accordingly, the complete sequence consists of (Fig 4):

(i) **Baseline walk (walk #1):**
  a The participant navigates a 25m flat surface loop at a natural/self-selected pace for 1-minute. Baseline cadence is determined.
(ii) **Cued walks (walks #2, #4, and #6 cue sequence varies by participant, as above)**
  b **Walk #2:**
    i The participant navigates the same 25m loop while allocated to a sequence (above) set at +10% of their baseline cadence (walk #1).
    ii Instructions to the participant: Walk to the beat of the cue.
    iii WO1 (Part 1: sitting and counting backwards followed by Part 2: 1-minute walk at self-selected pace)
  c **Walk #4:**
    iv The participant navigates the same 25m loop while allocated to a sequence (above) set at +10% of the baseline cadence.
    v Instructions to the participant: Walk to the beat of the cue.
    vi WO2 (Part 1: sitting and counting backwards followed by Part 2: 1-minute walk at self-selected pace)
  d **Walk #6:**
    vii The participant navigates the same 25m loop while allocated to a sequence (above) set at +10% of baseline cadence.
    viii Instructions to the participant: Walk to the beat of the cue.

### Outcome measures

#### Validation of gait characteristics.

This assesses the validity of CuePD to quantify PwPD gait characteristics against an established gold/reference-standard wearable (Opal). Gait characteristics include the mean, variability and asymmetry of temporal, spatial, and spatiotemporal characteristics (i.e., step time, stride time, stance time, swing time, stride length). Establishing the validity of CuePD in measuring gait characteristics is essential to accurately detect changes before and after cueing and to precisely tailor the administered cues to each individual’s physiological condition.

#### Impact of cueing.

This examines the ability of different personalised auditory cues to modify/retrain gait in PwPD. By comparing the effects of metronome beats, instrumental music, and vocal music on gait speed, stride-time variability, and stride length, this outcome seeks to identify which auditory cues most effectively enhance gait stability and performance. ${\text{Coefficient of variance of stride}-\text{time }(\text{CV}}_{\text{stride}-\text{time}})$ will be calculated as the standardised measure of the dispersion of stride times. This provides a more reliable measure of stride-time variability and has shown to be a significant indicator of gait instability and an independent predictor of fall risk among older adults and PwPD, underscoring its importance in clinical and rehabilitative settings [73]. Measuring changes in gait will be key to assessing CuePD as a gait retraining tool.

#### Acceptance and preference.

This focuses on understanding how PwPD accept and prefer personalised music cues over metronome cues for gait retraining. It involves gathering subjective feedback on the likeability, engagement, and perceived effectiveness of the music cues, especially in contrast with the metronome cues. Preference of musical cues will influence long-term adherence to a cueing intervention. This also includes investigating which cue is found to be the easiest to adhere to, as well as the results from the SUS questionnaire.

#### Adherence to cues and musicality.

Adherence will be treated both as a continuous variable (percentage change in cadence from baseline) and as a binary variable for group comparisons. The primary binary adherence definition is: adherent = ≥5% increase in cadence from baseline; non-adherent = < 5%. Mann–Whitney U tests will compare Gold-MSI between adherent and non-adherent participants. Participants with missing baseline or intervention-based cadence (e.g., wishing to withdraw or technology failure) will be excluded from binary adherence analyses and numbers excluded will be reported.

#### Sample size.

Sample size calculations were based on detecting changes in gait speed using a within-subject design. Assuming a standardised response mean (SRM) of 0.5 (moderate effect size) for gait speed, a two-sided significance level (α) of 0.01, and 90% power, approximately 54 participants would be required to detect a statistically significant change in gait speed for a single comparison (e.g., cued vs. non-cued walking). When accounting for the three cueing conditions and the associated adjustment for multiple (three) comparisons, we applied a Bonferroni correction resulting in an adjusted alpha of α (0.01/3 = 0.0033). That requirement increases sample size to approx. 55 participants. However, to allow for an anticipated attrition rate of up to 10%, we plan to recruit 60 participants in total. This sample size ensures adequate power to detect moderate cueing effects on gait speed, accommodates potential dropouts, and provides balanced representation across randomisation sequences.

#### Justification Summary.

A total sample size of 60 PwPD ensures robust and reliable results for both detecting significant changes in gait parameters and assessing the reliability of gait measurements across different auditory cues when compared to the reference standard.

### Statistical analysis

For the first primary objective, all gait characteristics derived from CuePD and reference will be compared using ICC_2,1_ (absolute agreement), a two-way random-effects model for single measures that evaluate the absolute agreement between raters. The level of agreement will be interpreted as: < 0.5 shows poor agreement, 0.5–0.75 moderate, 0.75–0.9 good and 0.9–1.0 excellent. PCC’s will be calculated to measure the linear correlation between the two sets of results. Correlation coefficients will be interpreted as follows: zero (0), weak (±0.1 to ±0.3), moderate (±0.4 to ±0.6), strong (±0.7 to ±0.9), and excellent/perfect (+1 or −1).

The median, mean, standard deviation (SD), minimum/min, maximum/max, and standardised response mean (SRM) of cadence, stride length, gait speed and CV_stride-time_ will be calculated. These descriptive statistics will provide context for interpreting changes and help to clarify the magnitude and consistency of cueing effects. SRM allows for assessment of responsiveness and comparison of results to other studies. The SRM will be interpreted as: > 0.8 large positive response, 0.5–0.8 moderate responsiveness, and <0.5 low responsiveness where positive values imply an improvement for cadence, gait speed and stride length but negative is an improvement for CV_stride-time_, i.e., reduces variability.

To evaluate the cueing modality adherence, the percentage change in cadence from the baseline walk to each cueing modality will be calculated for each participant. The most effective cueing modality will be identified as the one producing a change closest to 10%.

Model assumptions will be formally checked, where residual normality and normality of gait characteristics within each cue condition will be assessed using the Shapiro-Wilk test, and sphericity with Mauchly’s test. The Greenhouse-Geisser correction will be applied if sphericity is violated.

A two-way mixed ANOVA will be used to assesses the effects of cueing modality and walk order on outcomes, i.e., cadence, gait speed, stride length, and CV_stride-time_. Comparable to the referenced PD study, cueing modality will be modelled as a within-subject factor, while walk order as a between-subject factor to account for potential sequence effects. For each outcome, within-participant changes from baseline will be calculated using cue-specific difference scores. To account for differences in scale and statistical distribution among the outcomes, change scores will be computed using Equations (30)-(31):

- Percentage change of gait speed and stride length from baseline, calculated as:

$$\%\Delta =\frac{\text{Cue Value}-\text{Baseline Value}}{\text{Baseline Value}}\times \text{1}\text{0}\text{0}$$ (30)

- In contrast,${\text{ CV}}_{\text{stride}-\text{time}}$, a dimensionless ratio-based variable that may have a skewed distribution, change will be computed using the natural log-ratio of cue value to baseline:

$$\Delta_{log}=\text{log}\left(\text{Cue Value}+\in \right)-\text{log}\left(\text{Baseline Value}+\in \right)$$ (31)

where 𝜖 is a small constant to avoid the logarithm of zero. This transformation was chosen as ${\text{CV}}_{\text{stride}-\text{time}}$ is a ratio-based measure that can be highly skewed and exhibit unequal variances across conditions. Applying the *log* transformation to change scores helps to normalise the distribution and stabilise the variance, thereby supporting more reliable statistical comparisons of cueing effects on ${\text{CV}}_{\text{stride}-\text{time}}$ in this within-subjects design. Post-hoc comparisons between cueing modalities will be conducted using paired t-tests, with Bonferroni correction applied to account for multiple comparisons. Effect sizes will be reported using Cohen’s d.

If assumptions for normality are not met, a Friedman test will be used to assess within-subject differences across cueing conditions. Post-hoc Wilcoxon signed-rank tests, with Bonferroni correction, will be conducted only if the Friedman test is statistically significant. For between-subject effects (e.g., walk order), where normality assumptions are violated, the Kruskal–Wallis test will be used to assess group-level differences between walk-order groups. Interaction effects between cueing and walk order will be explored non-parametrically by conducting separate Friedman tests within each walk order group. Effect sizes for non-parametric tests will be calculated using the rank-biserial correlation coefficient, $\text{r}=\text{Z}/\sqrt{\text{N}}$, providing a measure of the magnitude of the effects observed.

For the three primary comparisons, we will control the familywise error rate using a Bonferroni adjustment. Given three cueing modalities, statistical significance (*p*) will be evaluated at a two-sided α = 0.01/3 = 0.0033. Effect sizes (i.e., SRM) and 95% confidence intervals will also be reported. Secondary and exploratory analyses (e.g., comparing cueing modalities to one another, examining washout effects, assessing stride-to-stride variability) will not be subject to multiplicity correction. For these analyses, a two-sided *p*-value of 0.05 will be considered statistically significant across all parametric and nonparametric tests.

To assess whether the first cue produced short-term residual effects, we will calculate changes in gait outcomes from baseline to each cued walk and from each cued walk to its subsequent washout walk (WO1 and WO2). For each cue exposure, a carryover score will be calculated as the difference between the change induced by the cue and the change observed during the corresponding washout period, reflecting retained cue effects. The approach enables an evaluation of carryover effects after each cued walk separately, thereby capturing any cumulative or progressive retention of cueing effects. Group differences based on initial cue type (i.e., counterbalanced sequence) will be evaluated using one-way ANOVA or the Kruskal–Wallis test, depending on normality assumptions [74–80].

To evaluate the relationship between musical sophistication and response to cueing, two sets of analyses will be conducted using Gold-MSI subscale scores. Assumption checks, including the Shapiro-Wilk test and Q-Q plot inspections, will be used to assess the distribution of change scores prior to both analyses. First, the association between Gold-MSI scores and cue adherence (defined as the absolute deviation from a + 10% target increase in cadence) will be assessed using Spearman’s rank correlation coefficient, with Bonferroni correction for multiple comparisons across subscales. Second, associations between Gold-MSI scores and gait outcome improvements (percentage change in gait speed and stride length, and log-ratio change in ${\text{CV}}_{\text{stride}-\text{time}}$) will also be tested using Spearman’s correlation, with Bonferroni correction.

### Data management and availability

The study adheres to the General Data Protection Regulation (GDPR) and the Data Protection Act 2018, which mandate that data be de-identified as soon as reasonably possible. All data collected in this study will be anonymised, with each participant assigned a unique study code. Personal information will be securely stored in locked filing cabinets at the principal investigators (AG) office at Northumbria University and will only be accessible to the research staff directly involved in the study. Data will be entered into an electronic database using the unique study codes and securely stored on a password-protected computer. Any significant protocol changes during the study will be reported to the trial registry. The de-identified participant data that support the findings of this study will be available from the Northumbria University Research Ethics Committee. Access requires approval from the Committee and a signed data-sharing agreement. Requests should be sent to the corresponding author, AG (alan.godfrey@northumbria.ac.uk), who will coordinate requests with the Committee.. The findings will be shared through academic outputs, including national and international conferences and publications.

## Discussion

The protocol outlined for the validation and exploration of CuePD, a proposed approach to scale assessment and retraining in gait rehabilitation for PwPD. It adopts a novel approach by integrating personalised auditory cues through pervasive smartphone technology. This method diverges significantly from traditional rehabilitation strategies by emphasising a user-centric approach to enhance engagement and encourage long-term use in PwPD.

### Clinical validation

Previous CuePD studies have established validity within non-clinical cohorts [61,62] but this protocol aims to establish the validity of temporal, spatial, and spatiotemporal gait characteristics in PwPD compared to an established gold/reference-standard wearable. Establishing robust validity is essential to ensure reliable measurements, which are critical for the effective administration of targeted and personalised cueing interventions that often lead to more significant improvements in gait [81,82].

### Primary objectives: Validity and evaluating the effects of personalised auditory cues on gait

The impact each personalised auditory cue has on participants’ stride length, gait speed, and${\text{ CV}}_{\text{stride}-\text{time}}$ are a significant exploratory interest. These metrics, in particular${\text{ CV}}_{\text{stride}-\text{time}}$, where a decrease is known to reduce the risk of falling, have indicated variable effects among PwPD in other cueing-related studies, particularly where music cueing is not personalised [9,44,73]. The findings from this study will establish a baseline understanding of their (i) validity (from an app and smartphone worn on the lower back) and (ii) whether personalised cues can consistently reduce stride variability across a broader range of participants.

Observed gait responses will be interpreted in the context of sequence-conditional effects (cue order, exposure, and adaptation over time), and not as definitive evidence of causal superiority of one cueing condition over another. Analyses of gait variability and asymmetry will be interpreted cautiously, as these measures typically exhibit greater dispersion and may be underpowered (as sample size of based on gait speed). Emphasis will be placed on estimating effect sizes and examining consistency of responses across conditions, rather than on formal hypothesis testing.

### Exploratory objectives: preference, usability, and musicality

Exploratory objectives of the protocol also focus on the user experience and the clinical efficacy of the personalised auditory cues provided by CuePD. A significant part of this exploration involves understanding the preferences of PwPD for engaging with personalised music cues during routine walking exercises, compared to traditional metronome cues which are often perceived as monotonous and less engaging. This preference for music could play a crucial role in enhancing patient engagement and adherence to the rehabilitation process. Finally, the study explores how well patients’ musicality correlates with their ability to synchronise with the auditory cues. If no relationship is found, this would indicate that the success of music cueing-based interventions does not depend on a patient's inherent musical skills, potentially broadening the applicability of such treatments to a wider demographic of PwPD.

### Usability and practical implications

Usability is a critical aspect of this protocol, as the practical application of CuePD depends on its potential ease of integration into daily routines and the overall acceptance of the methods proposed by PwPD. This feedback will be evaluated using the SUS questionnaire, to substantiate that utilising a smartphone-based system to provide gait analysis and rehabilitation could be a more appealing and effective option, presenting it as a viable and accessible alternative to traditional methods.

### Potential limitations and future considerations

This study will be conducted in a controlled laboratory setting, which allows for precision in measuring gait characteristics. However, the lab environment may not fully replicate real-world conditions, where environmental variables and day-to-day changes in motor symptoms may influence gait. Equally, the short testing duration in the lab does not provide clarity on long-term adherence in daily life. Moreover, although the study will use two sources for recruiting participants, there may be some bias introduced due to the use of the DeNDRoN case register as some participants may be more likely to be already motivated to take part in research, may have higher education or health literacy.

Future studies will consider validating CuePD in home and/or community-based settings to assess its feasibility and effectiveness outside of the lab. Accordingly, examining CuePD beyond the lab will provide insights to long-term adherence. Additionally, the study focuses on PwPD who can walk unaided, which may limit the generalisability of the findings to those with more advanced PD and/or those prone to episodes of freezing. Expanding future research to include these populations will be important to ensure CuePD’s broad applicability.

## Conclusion

The CuePD protocol will provide evidence for the validity and usability of a smartphone based approach to gait rehabilitation in PwPD. Its primary objectives are to validate and evaluate the effects of personalised auditory cues on gait characteristics, establishing a rigorous framework for assessing changes in gait speed, stride length, and stride time variability. This study will also validate the accuracy of the CuePD system in assessing detailed gait characteristics. CuePD may offer a potentially more effective, engaging, and accessible alternative to traditional gait rehabilitation methods. This could significantly enhance the quality of life for PwPD, encouraging more consistent and long-term engagement in rehabilitation activities.
